# Arterial Stiffness and Early Cardiac Dysfunction in Type 2 Diabetes Mellitus: A Potential Role for 25 OH Vitamin D3 Deficiency

**DOI:** 10.3390/medicina61081349

**Published:** 2025-07-25

**Authors:** Laura Maria Craciun, Florina Buleu, Stela Iurciuc, Daian Ionel Popa, Gheorghe Nicusor Pop, Flavia Goanta, Greta-Ionela Goje, Ana Maria Pah, Marius Badalica-Petrescu, Olivia Bodea, Ioana Cotet, Claudiu Avram, Diana-Maria Mateescu, Adina Avram

**Affiliations:** 1 Department of Cardiology, “Victor Babes” University of Medicine and Pharmacy, 300041 Timisoara, Romania; laura.craciun@umft.ro (L.M.C.); anamaria.pah@umft.ro (A.M.P.); marius.badalica-petrescu@umft.ro (M.B.-P.); 2 Doctoral School, “Victor Babes” University of Medicine and Pharmacy, 300041 Timisoara, Romania; daian-ionel.popa@umft.ro (D.I.P.); olivia-maria.bodea@umft.ro (O.B.); ioana.cotet@umft.ro (I.C.); diana.mateescu@umft.ro (D.-M.M.); 3 Research Center for Medical Communication, “Victor Babes” University of Medicine and Pharmacy, 300041 Timisoara, Romania; 4 Center for Modeling Biological Systems and Data Analysis (CMSBAD), “Victor Babes” University of Medicine and Pharmacy, 300041 Timisoara, Romania; pop.nicusor@umft.ro; 5 Clinical Hospital CFR Timisoara, 300175 Timisoara, Romania; flaviagoanta@gmail.com; 6 Advanced Cardiology and Hemostaseology Research Center, “Victor Babes” University of Medicine and Pharmacy, No. 2 Eftimie Murgu Square, 300041 Timisoara, Romania; barbulescu.greta@umft.ro; 7 Department I of Nursing, University Clinic of Clinical Skills, “Victor Babes” University of Medicine and Pharmacy, No. 2 Eftimie Murgu Square, 300041 Timisoara, Romania; 8 Department XVI-Balneology, Medical Recovery and Rheumatology, “Victor Babes” University of Medicine and Pharmacy, 300041 Timisoara, Romania; avram.claudiu@umft.ro; 9 Department of Internal Medicine I, “Victor Babes” University of Medicine and Pharmacy, 300041 Timisoara, Romania; avram.adina@umft.ro

**Keywords:** type 2 diabetes mellitus, arterial stiffness, vitamin d, 25(OH)D3, intima–media thickness, pulse wave velocity, global longitudinal strain, early cardiac dysfunction

## Abstract

*Background and Objectives*: Type 2 diabetes mellitus (T2DM) is associated with subclinical cardiovascular changes, such as increased arterial stiffness and myocardial dysfunction. Vitamin D deficiency has been recognized as a potential contributing factor to vascular disease; however, its impact on early cardiac changes associated with T2DM remains poorly understood. Our aim was to evaluate the association between serum levels of 25-hydroxyvitamin D3 [25(OH)D3], arterial stiffness, and left ventricular global longitudinal strain (LV GLS) in patients with T2DM who do not have a clinically evident cardiovascular disease. *Material and methods:* This cross-sectional study evaluated the carotid intima–media thickness (IMT), aortic pulse wave velocity (PWVao), LV GLS, and serum 25(OH)D3 levels in patients diagnosed with T2DM (*n* = 65) compared to healthy control subjects (*n* = 55). Independent predictors of arterial stiffness were identified by a multivariate logistic regression analysis. *Results:* Patients with T2DM showed a significant increase in IMT and PWVao, a reduction in LV GLS, and low levels of 25(OH)D3 compared to subjects in the control group (all *p* < 0.05). Both vitamin D deficiency and T2DM were found to be independently associated with an increased arterial stiffness, with odds ratios of 2.4 and 4.8, respectively. A significant inverse relationship was identified between 25(OH)D3 levels and markers of arterial stiffness, as well as LV GLS, suggesting a possible association between the vitamin D status and the early onset of cardiovascular dysfunction. *Conclusions:* Patients with T2DM show early signs of heart and blood vessel problems, even with an ejection fraction that remains within normal limits. There is a significant correlation between vitamin D deficiency and increased arterial stiffness, along with impaired LV GLS, indicating its possible involvement in cardiovascular complications associated with diabetes. These findings support the utility of integrating vascular, myocardial, and vitamin D assessments in early cardiovascular risk stratification for T2DM patients.

## 1. Introduction

Patients who have been diagnosed with type 2 diabetes mellitus (T2DM) face a markedly elevated risk of macroangiopathy when compared to individuals without diabetes [1]. Furthermore, diabetic vascular disease is associated with a two-to-four-fold increase in the rates of coronary artery disease (CAD) and stroke, along with a two-to-eight-fold rise in the probability of developing heart failure [2]. The heightened cardiovascular risk present in diabetic patients cannot be solely explained by traditional risk factors, which encompass age, sex, hypertension, dyslipidemia, and smoking. Given that elevated blood pressure remains one of the most significant cardiovascular risk factors [3], it is essential to consider a comprehensive strategy to deliver an optimal personalized antihypertensive medical approach for these patients as a key objective [4]. This increased risk appears to be related to endothelial dysfunction and inflammation [5]. Consequently, it is plausible that other non-traditional risk factors may be of considerable importance for people with T2DM. 

Widely recognized for its importance in calcium and bone metabolism, vitamin D has evolved into a multifunctional hormone with pleiotropic effects on several body systems. In addition to its established roles, vitamin D has demonstrated immunomodulatory, anti-inflammatory, and antioxidant properties, suggesting a possible link to the pathogenesis of atherosclerosis [6,7]. Given the widespread prevalence of vitamin D deficiency globally and the alarming frequency of cardiovascular events, exploring the role of vitamin D in atherosclerosis has become increasingly important [7,8].

The measurement of the pulse wave velocity (PWV) is a reliable, validated, and straightforward indicator of arterial stiffness. In essence, arterial blood pressure configuration results from a combination of the forward pressure wave produced by ventricular contraction and a-wave reflected from a more distal location. The aortic PWV is widely acknowledged as the gold standard for evaluating arterial stiffness, supported by numerous studies [9,10]. It is important to note that a high prevalence of vitamin D deficiency is observed among individuals with T2DM, which is significantly associated with the extent of atherosclerosis, as determined by the carotid intima–media thickness (IMT) [11] and PWV [12].

In a study conducted by Cheng et al. involving 95 patients (average age 62 ± 9 years, with 58% being female) diagnosed with T2DM and lacking a history of CAD, it was found that vitamin D deficiency is independently linked to an impaired left ventricular global longitudinal strain (LV GLS). The authors concluded that vitamin D deficiency could play a role in the onset of myocardial dysfunction among these individuals [13]. Although earlier research has investigated the links between vitamin D deficiency, arterial stiffness, and myocardial dysfunction, limited attention has been given to their possible interconnection. This study represents the first attempt to simultaneously assess serum vitamin D levels, arterial stiffness, and subclinical myocardial impairment—measured through GLS—in asymptomatic adults with diabetes mellitus—particularly those without clinical symptoms of heart disease—and healthy subjects in the control group. This innovative integrative methodology has the potential to reveal subtle cardiovascular changes before the onset of an overt disease.

Therefore, our objectives included correlating 25(OH)D3 levels with indicators of arterial stiffness, assessed by the PWV and IMT, along with left ventricular function parameters in patients with type 2 diabetes mellitus, compared with healthy individuals.

## 2. Materials and Methods

### 2.1. The Study Design and the Selection of Participants

This case–control study was conducted at the Institute of Cardiovascular Diseases in Timișoara, Romania, involving 65 patients aged over 18 years with type 2 diabetes mellitus (T2DM group) without complications, who were admitted to the clinic over the course of one year. These patients were selected from a total of 504 hospitalized individuals diagnosed with type 2 diabetes mellitus. Although all participants underwent diagnostic coronary angiography, only those who did not undergo revascularization procedures were included in this study, according to their individual medical documents.

Patients diagnosed with T2DM were assessed alongside a control cohort comprising 55 healthy individuals of equivalent age, the healthy group. This group was selected concurrently from a database of nearly 700 subjects evaluated for cardiovascular risk factors by family physicians affiliated with our clinic. The control participants were free from cardiovascular diseases, such as coronary artery disease, peripheral artery disease, carotid artery disease, heart failure, or stroke, and they also did not have any inflammatory diseases, active infections, or known neoplasms. Furthermore, within this group, systemic blood pressure values were observed without administering antihypertensive medications, fasting blood glucose levels were kept below 100 mg/dL, and lipid profile parameters remained within normal ranges.

This study adhered to the Declaration of Helsinki and received approval from the Ethics Committee at the Institute of Cardiovascular Diseases in Timișoara, Romania (approval no. 1265). Informed consent was obtained from all participants involved in the research. This study was not registered due to its observational nature.

### 2.2. Clinical and Biochemical Evaluation

The clinical and biochemical evaluation were conducted for all patients. Data on age, sex, the state of diabetic microangiopathy or macroangiopathy, state of hypertension, state of dyslipidemia, and smoking status were recorded. A standard objective examination was carried out measuring systolic as well as diastolic blood pressure and body mass index (BMI). The European Guidelines for the prevention of cardiovascular disease in clinical practice were followed in measuring blood pressure. A standard tensiometer (Riester, Jungingen, Germany) was utilized, accompanied by a suitable cuff for each subject’s arm. Measurements were performed on both arms, with the highest reading being recorded. Body weight (kg) was assessed using a mechanical scale, while height (m) was determined with a metal tape measure (Fazzini, Vimodrone, Italy). The BMI is calculated by weight in kilograms divided by height in meters squared; that is BMI = weight (kg) ÷ height2 (m^2^). For the measurement of fasting blood glucose, hexokinase testing was performed using Siemens Dimension RXL-MAX, reagents from Dade Behring, Erlangen, Germany. Triglycerides, total cholesterol, and LDL and HDL-cholesterol fractions were measured using photometric techniques (Siemens Dimension RXL-MAX, utilizing reagents from Dade Behring, the same manufacturer). The determination of creatinine levels in serum was performed via the Jaffe manual method without the need for deproteinization. The estimated glomerular filtration rate (eGFR) was calculated using the MDRD formula: eGFR = 186 × (Creatinine/88.4) − 1.154 × Age − 0.203 × 0.742 for females. Hemoglobin A1c levels were assessed immunoturbidimetrically, adhering to the standards established by the Diabetes Control and Complications Trial (DCCT) [14] and certified by the National Glycohemoglobin Standardization Program (NGSP) [15]. According to the American Diabetes Association (ADA), a result between 4.8% and 5.6% is considered normal, while results between 5.7% and 6.4% indicate a high risk for developing diabetes mellitus (DM); a value of 6.5% or higher is indicative of type 2 diabetes mellitus.

Patients with T2DM received their diagnosis before being admitted to the hospital, which aligns with the consensus report from the ADA [16].

#### 2.2.1. Hydroxyvitamin D_3_ Analysis

The serum levels of 25(OH)D3 were determined using the DIAsource 25-OH Vitamin D Total Elisa 90 kit, produced by DIAsource Immunoassays SA, located in Louvain-la-Neuve, Belgium. Two samples with varying concentrations—one low and one high—were analyzed to evaluate both reproducibility and accuracy. These concentrations were assessed in duplicate over 10 distinct runs to establish reproducibility and within a single run to gauge precision and repeatability. The measurements were calibrated and validated using the Free 25 OH Vitamin D serum control, as specified in the application notes provided by the manufacturer. The blank (LOB) limit was set at 2.012 ng/mL. The limit of detection (LOD) was calculated by deducting 1.645 times the standard deviation of a low-concentration sample, tested across 10 different runs, from the LOB, yielding an LOD of 3.035 ng/mL. The interpretation of the measured values is categorized as follows: levels of 30 ng/mL or above are deemed sufficient, levels between 21 ng/mL and 29 ng/mL are classified as insufficient, mild deficiency is indicated by levels ranging from 10 ng/mL to 20 ng/mL, moderate deficiency corresponds to levels exceeding five ng/mL but falling below ten ng/mL, and levels below five ng/mL define severe deficiency [8,17]. 

Consequently, to ensure that the serum 25(OH)D3 level remained uninfluenced during the study period, this study exclusively included patients from whom blood samples were collected between 1 March and 1 November. Additionally, none of the patients reported exposure to excessive sunlight, as advised before inclusion.

#### 2.2.2. The Ultrasound Evaluation of the Heart and Carotid Arteries

All subjects underwent transthoracic echocardiography utilizing the GE Vivid 9 ultrasound system from GEMS Ultrasound in Tirat Carmel, Israel. Each patient underwent color flow, Doppler, M-mode, and 2D imaging studies. The M-mode ultrasound provided dynamic images of all structures intersecting the beam over time, facilitating precise measurements of cardiac structures.

**Figure 1:**
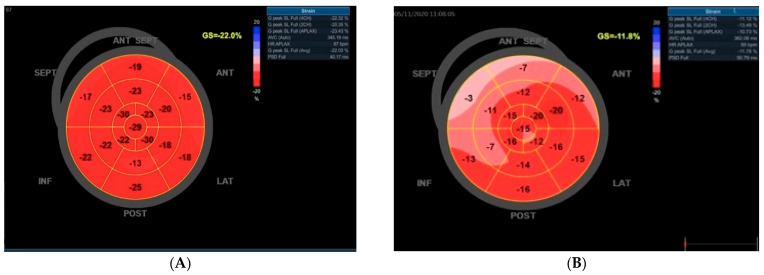
An illustration of bull’s eye maps utilizing foreign echocardiography. (**A**) In a normal subject devoid of cardiovascular risk factors and exhibiting a normal coronary angiography, a typical bull’s eye map is observed, reflecting a preserved global left ventricular longitudinal strain of −22.0%. (**B**) Conversely, a diabetic patient displays a notable reduction in the left ventricular global longitudinal strain, recorded at −11.8% on the bull’s eye map. For both subjects, the coronary angiography revealed no significant obstructive coronary artery disease.

Two-dimensional images were obtained from the long and short axis parasternal views corresponding to the three standard apical perspectives: 2-chamber, 3-chamber, and 4-chamber results. The measurements of left ventricular chamber size and wall thickness were conducted by the Recommendations for Cardiac Chamber Quantification by Echocardiography in Adults: An Update from the American Society of Echocardiography and the European Association of Cardiovascular Imaging [18]. Transmitral blood flow was recorded at the four-chamber apical view with pulsed wave Doppler. E-wave rate, maximum mitral late filling velocity A, E/A ratio, and E-wave deceleration time (DT) were measured. Tissue Doppler was applied in the septum and lateral; e’ was calculated for the calculation of the E/e’ ratio. Speckle-tracking analysis was determined by a dedicated wall motion tracking system from GE Vingmed Ultrasound AS, Horten, Norway. Peak longitudinal systolic strain values were assessed in the basal, middle, and apical regions across six standard segments of the left ventricle, facilitating precise segmental analysis alongside the evaluation of global longitudinal strain depicted in the bull’s eye model in Figure 1, which employs a 17-segment framework for the LV. The region of interest (ROI) undergoes visual assessment and may be manually adjusted to guarantee accurate speckle-tracking. The normal range for global longitudinal strain (GLS) is 18% or lower (i.e., more negative). In comparison, values of −16% or higher (i.e., less negative) are considered abnormal, with a range of −16% to −18% being classified as borderline [19]. Patients exhibiting more than three inaccurately tracked segments due to insufficient image quality were excluded from subsequent analysis. Figure 1 provides examples of speckle-tracking analysis from patients participating in the study.

Doppler carotid echography was performed on all patients participating in the study. To identify carotid atheromatosis, we conducted measurements of intima–media thickness, with values of less than 0.9 mm deemed to be within the normal range [20].

#### 2.2.3. Measurement of Arterial Stiffness

Manufactured by TensioMedKft, located in Budapest, Hungary, the MedexpertArteriograph device, version 3.0.0.3, was employed to perform assessments of carotid–femoral pulse wave velocity. This instrument facilitated the measurement of central pressure, the augmentation index (Aix), and the pulse wave propagation velocity within the aorta (PWVao). Before measurements, subjects underwent a minimum resting period of 10 min in a tranquil environment, positioned supinely. The same examiner conducted repeated measurements by the methodology specified in the expert consensus document concerning arterial stiffness [9,21]. For a minimum duration of 3 h before the measurements, the consumption of smoking products and any foods or beverages containing caffeine was prohibited. Additionally, alcohol intake was ceased at least 10 h prior to the investigation. Similarly to blood pressure, both arterial diameter and arterial stiffness exhibit circadian variation, with increases occurring during sleep. Consequently, patients were instructed not to fall asleep while these parameters were being assessed. During the measurement process, patients refrained from speaking.

### 2.3. Statistical Analysis

Continuous variables were expressed in terms of medians and quartiles (Q1, Q3) and compared using the Mann–Whitney U test. Categorical variables were summarized by absolute and relative frequencies (%) and analyzed using the Chi-square test.

To explore the potential connection between vitamin D levels and other continuous variables, Pearson correlation coefficients were calculated. Multicollinearity was evaluated using Pearson correlations, and a Variance Inflation Factor (VIF) value below 5 was considered acceptable. To identify independent factors associated with arterial stiffness, measured by pulse wave velocity (PWV), we employed multivariate logistic regression. Model selection was performed using the Akaike Information Criterion (AIC), as implemented by the ‘glmulti package’, to determine the most parsimonious model.

Post hoc power calculations (α = 0.05, two-tailed) showed >90% power to detect a large effect size (Cohen’s d ≈ 0.8) for key comparisons like vitamin D levels and a 60–70% power for subgroups.

Data analysis was conducted using R version 4.4.0 [22]. The following R packages were used: corrplot v0.92, glmulti v1.0.8, rstatix v0.7.2, ggpubr v0.6.0, and car v3.1-3.

A *p*-value < 0.05 was considered the threshold for statistical significance.

## 3. Results

### Patient Population and Characteristics

The clinical, biochemical, and demographic features of the patients are summarized in Table 1. There was no difference between healthy and DM patients in the age and sex distribution. The heart rate and diastolic blood pressure were also similar between the two groups; however, the BMI and systolic blood pressure were significantly higher in the DM group. The fasting plasma glucose and HbA1c were significantly higher in diabetic participants (*p* < 0.001). The lipid profile (LDL and triglycerides) was significantly higher in patients with diabetes mellitus. No statistically significant difference was observed in the smoking prevalence between groups (*p* = 0.3), and smoking was not significantly correlated with serum 25(OH)D3 levels in either group, with the stratified analysis showing no statistically significant difference in vitamin D levels regardless of the smoking status (22.28 vs. 23.56 ng/mL; *p* = 0.29). There were no differences observed between the groups with respect to creatinine and the eGFR. A significantly lower value for 25(OH)D3 is shown for the diabetic group, as well as a higher incidence of vitamin D deficiency (Table 1).

In patients with type 2 diabetes mellitus, the values for the LAD, IVS, LV PW, and E/e′ were found to be significantly elevated compared to those of normal controls (*p* < 0.001). Conversely, the LV GLS value among T2DM patients was significantly reduced when compared to normal controls (*p* < 0.001). No significant differences were observed in the LV EF (*p* = 0.080), LV EDD (*p* = 0.002), LAV (*p* = 0.023), and e′ levels between the normal controls and T2DM patients (*p* = 0.9). Additionally, both measures of arterial stiffness were significantly diminished in individuals with T2DM (*p* < 0.001) (Table 2).

**Figure 2:**
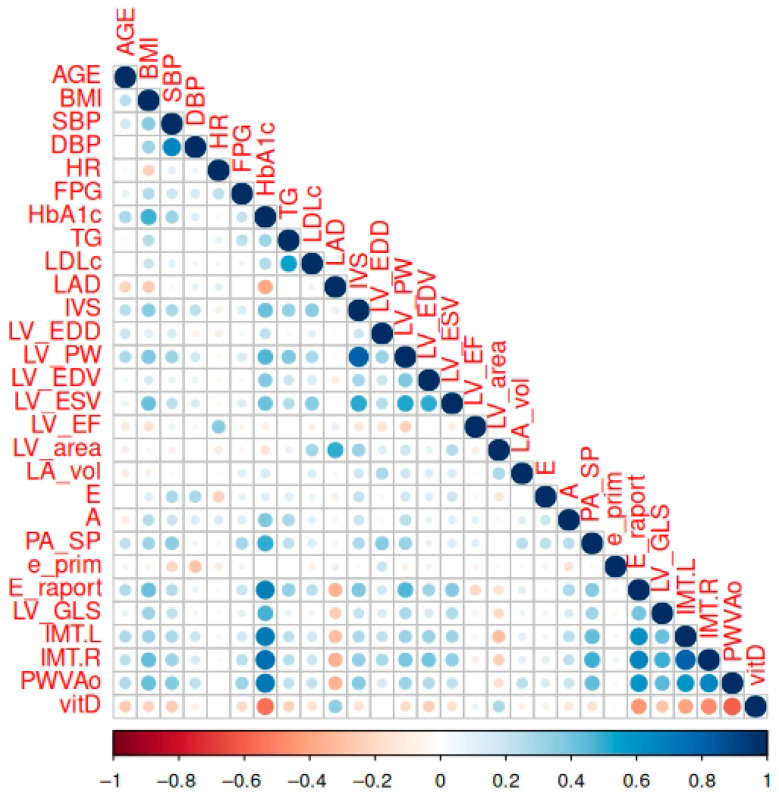
The correlation matrix between the studied parameters. A correlation is significant at *p* < 0.01.

The correlation analysis revealed several strong associations among cardiovascular and metabolic parameters. Notably, left ventricular dimensions and volumes (e.g., LV_EDD, LV_EDV, LV_ESV) showed high positive correlations (*r* > 0.8), reflecting structural interdependence, while the LV ejection fraction (LV_EF) was inversely correlated with both end-diastolic and end-systolic volumes (*r* ≈ −0.7 to −0.8), which is consistent with the impaired systolic function in dilated ventricles. The bilateral carotid intima–media thickness (IMT.L and IMT.R) was highly correlated (*r* ≈ 0.9), indicating systemic vascular changes. Additionally, moderate to strong associations were observed among pulmonary artery pressures and vascular stiffness measures, while metabolic variables (e.g., BMI, HbA1c, TG) formed a modestly correlated cluster. In contrast, variables such as vitamin D and heart rates displayed a minimal correlation with most cardiovascular metrics. These findings underscore latent structural and functional constructs within the dataset and suggest the need for a dimensionality reduction when developing multivariate models (Figure 2).

**Figure 3:**
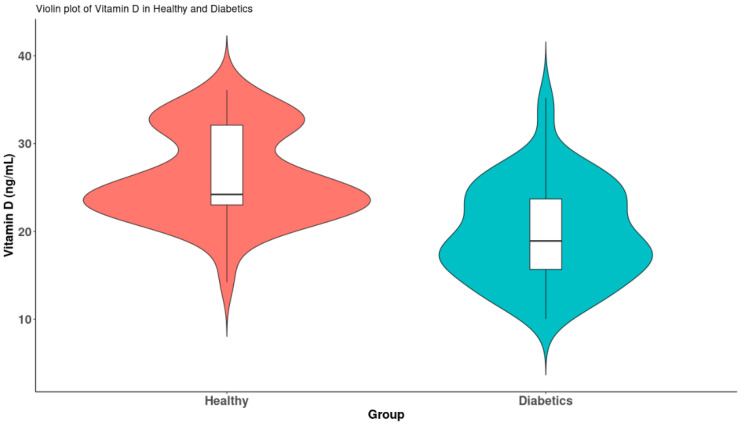
Violin plots of vitamin D levels between the two groups.

The mean values of 25(OH)D3 were significantly lower in diabetes mellitus type 2 patients compared to the healthy group (*p* < 0.001) (Figure 3).

**Figure 4:**
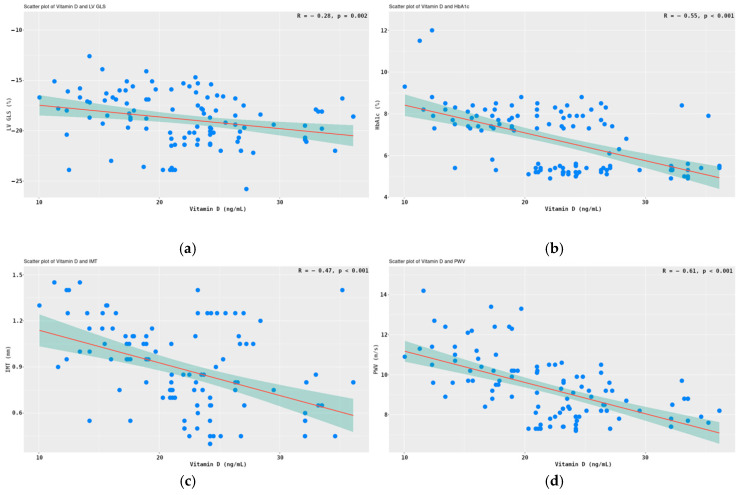
Correlations between 25(OH)D3 and LV GLS (**a**), HbA1c (**b**), IMT (**c**), and PWVao (**d**). Legend: Pearson correlation test, 95% CI shaded in teal color.

There was a significant negative correlation between 25(OH)D3 and LV GLS (*r* = −0.28, *p* =0.002) (Figure 4a). Vitamin D levels were also inversely related to the glycated hemoglobin; *r* =-0.55 and *p* < 0.001 (Figure 4b). Strong negative correlations of the parameters of arterial stiffness with vitamin D were observed. Furthermore, a correlation exists between 25(OH)D3 and the IMT (*r* = −0.47, *p* < 0.001), as illustrated in Figure 4c. Additionally, a second correlation can be observed between 25(OH)D3 and LV GLS (*r* = −0.61, *p* < 0.001), depicted in Figure 4d.

**Figure 5:**
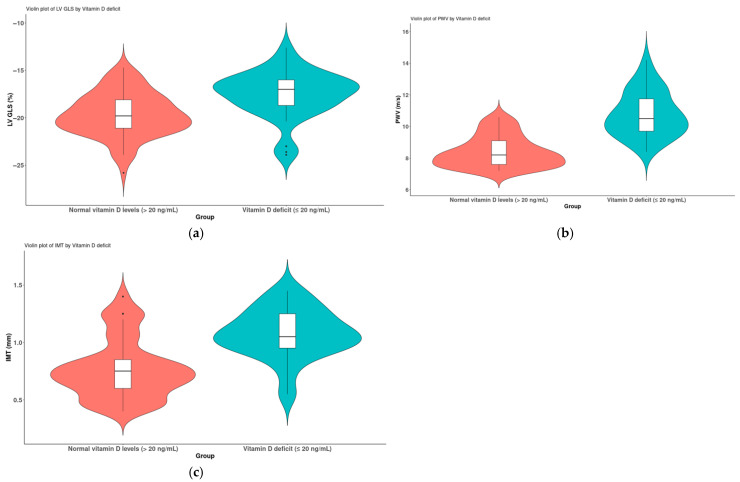
A comparison of patients diagnosed with diabetes mellitus (*n* = 65) based on their vitamin D levels (>20 ng/mL and ≤20 ng/mL); (**a**) LV GLS, (**b**) PWV and (**c**) IMT.

The 65 patients diagnosed with diabetes mellitus were categorized into two subgroups based on their vitamin D levels, specifically those with levels of ≥20 ng/mL and those with levels of <20 ng/mL. Given that the values did not conform to a normal distribution, comparisons between the two subgroups were conducted using the non-parametric Mann–Whitney U test. The results are illustrated in Figure 3. A significant reduction in LV GLS was observed in association with the vitamin D deficiency (*p* < 0.001) (Figure 5a). Additionally, indicators of vascular stiffness, the PWVao and IMT, were found to be significantly elevated in cases of vitamin D deficiency (*p* < 0.001) (Figure 5b,c).

We employed a multivariate logistic regression analysis to determine independent risk factors associated with arterial stiffness (IMT ≥ 0.9 mm or PWV ≥ 9 m/s). The results, including the odds ratio and 95% confidence interval, are detailed in Table 3. The factors identified as independently increasing the risk of arterial stiffness include diabetes mellitus, which elevates the risk of endothelial dysfunction by a factor of 4.8, and vitamin D deficiency, which increases the risk by a factor of 2.4.

## 4. Discussion

In the present study, we observed significant changes marked by increased IMT and PWVao values, both established indicators of arterial stiffness—along with reduced serum levels of 25(OH)D3, a biomarker increasingly associated with vascular health. These alterations were evident in individuals with type 2 diabetes mellitus who had no prior history of cardiovascular disease, when compared to age- and sex-matched healthy controls. While numerous studies have demonstrated associations between 25(OH)D3 levels and coronary atherosclerosis [23], as well as vascular endothelial dysfunction in diabetic patients [1], there is a considerable lack of research investigating the association between arterial stiffness biomarkers and global longitudinal strain—a sensitive indicator of subclinical myocardial dysfunction—particularly among the T2DM patient population [24].

A major strength of our study is its innovative, integrative design. It is, to our knowledge, the first to concurrently investigate the vitamin D status, arterial stiffness, and GLS in asymptomatic individuals. These parameters are often studied in isolation but are rarely, if ever, examined together. By synthesizing these components, our findings offer new insights into early cardiovascular remodeling that may be driven by vitamin D deficiency. This triad—25(OH)D3, arterial stiffness, and GLS—has not previously been evaluated in a single study among asymptomatic T2DM patients, and our results suggest the presence of a subclinical mechanism through which vitamin D may influence cardiovascular health well before a clinical disease becomes evident.

Prior investigations conducted by Liu et al. [25] and Wang et al. [24] established that a reduced LV GLS in patients with type 2 diabetes mellitus who have no history of cardiovascular disease serves as a predictor of future cardiovascular events.

Our results are consistent with these findings: individuals with T2DM had significantly lower (i.e., more positive) LV GLS values compared to healthy subjects in the control group, despite maintaining a preserved left ventricular ejection fraction (*p* = 0.080). This observation indicates the presence of subclinical myocardial dysfunction, potentially related to pathological alterations in the diabetic myocardium. In particular, our study highlights the link between longitudinal variations in the arterial stiffness and left ventricular morphology and function in patients with T2DM without an overt cardiovascular disease. A significant relationship was identified between the progression of arterial stiffness and impaired ventricular remodeling, even at an early stage, before any visible decline in the ejection fraction. These findings underscore the clinical importance of vascular–ventricular coupling in the early development of myocardial dysfunction associated with T2DM.

In T2DM, chronic hyperglycemia leads to molecular and metabolic alterations within cardiomyocytes, negatively impacting coronary microcirculation. The resultant hypoxia and ischemia play a role in the development of myocardial hypertrophy, perivascular fibrosis, increased ventricular stiffness, and both systolic and diastolic dysfunction [26]. Epidemiological research indicates a correlation between vitamin D deficiency and impaired glycemic control, as well as the occurrence of type 2 diabetes mellitus [27]. Our group of diabetic patients demonstrated significantly higher levels of glycated hemoglobin (*p* < 0.001) and fasting plasma glucose (*p* = 0.004) when compared to healthy subjects.

Vitamin D deficiency is frequently observed in individuals with T2DM [1]. Notably, a 10 ng/mL decrease in serum 25(OH)D3 levels has been associated with a two-fold increase in all-cause mortality. In a large longitudinal study, vitamin D deficiency emerged as the most significant predictor of both primary cardiovascular outcomes and overall mortality during a 5.6-year follow-up period involving high-risk T2DM patients [28]. Our research similarly identified a robust inverse relationship between levels of 25(OH)D3 and early indicators of atherosclerosis (IMT and PWVao) in patients with T2DM.

Further supporting this, the previous research demonstrated that arterial stiffness in T2DM is independently associated with vitamin D deficiency, alongside factors such as age and blood pressure [12]. A study involving 314 patients diagnosed with T2DM employed a multiple linear regression analysis to reveal that serum 25(OH)D3 levels were inversely and independently correlated with the carotid IMT (β = −0.009, *p* < 0.01). Additionally, a logistic regression analysis affirmed an independent association with the presence of carotid plaques (OR = 0.95; 95% CI: 0.92–0.98, *p* = 0.004) [29].

In order to ascertain independent risk factors associated with arterial stiffness, defined as either an intima–media thickness of ≥0.9 mm or a pulse wave velocity of ≥9 m/s, we performed a multivariate logistic regression analysis. The odds ratios (ORs) and corresponding 95% confidence intervals (CIs) were computed, with the findings displayed in Table 3. The analysis revealed that both diabetes mellitus and vitamin D deficiency serve as independent risk factors for arterial stiffness. Specifically, diabetes mellitus was linked to a 4.8-fold increase in risk (OR = 4.8, 95% CI: 2.53–8.10), whereas vitamin D deficiency was associated with a 2.4-fold increase in risk (OR = 2.4, 95% CI: 0.28–5.03). In a cohort study involving 560 adults, individuals in the lowest quartile of 25(OH)D3 (<20 ng/mL) exhibited an adjusted OR of 2.04 (95% CI: 1.26–3.30) for an aortic PWV ≥ 9 m/s when compared to those in higher quartiles [30].

In our cohort, the smoking status was also recorded, which is a recognized cardiovascular risk factor analyzed in the literature in connection with vitamin D deficiency. No statistically significant difference was observed in the smoking prevalence between groups (*p* = 0.3), and smoking was not significantly correlated with serum 25(OH)D3 levels in either group, with the stratified analysis showing no statistically significant difference in vitamin D levels regardless of the smoking status (22.28 vs. 23.56 ng/mL; *p* = 0.29). Numerous clinical and population-based studies indicate that smokers demonstrate significantly lower serum levels of 25-hydroxyvitamin D in comparison to non-smokers, irrespective of factors such as age, the body mass index, sun exposure, or comorbidities. This negative correlation seems to be influenced by various mechanisms, including the pro-inflammatory and oxidative stress effects induced by tobacco smoke, which may disrupt the enzymatic conversion of vitamin D in the liver and kidneys and potentially hinder the expression of vitamin D receptors in peripheral tissues. Additionally, lifestyle factors associated with smoking—such as a diminished dietary vitamin D intake, reduced physical activity, and limited sun exposure—exacerbate this deficiency. A meta-analysis conducted by Yang et al. found that current smokers had significantly lower vitamin D levels than non-smokers (*p* < 0.001), regardless of existing disease states [31]. In a study with 300 participants, most of them had either insufficient or deficient vitamin D levels (84%). Tobacco cigarette-smoking elders had a significantly low serum vitamin D level (both deficiency and insufficiency) as compared to the non-smoker group (*p* < 0.05) [32]. Taken together, this information implies that tobacco consumption may represent a modifiable risk factor for vitamin D deficiency, which could have significant consequences for bone health, the immune system performance, and the cardiovascular risk in populations that are not diabetic.

The clinical implications of our study findings are significant. The noted decreases in the left ventricular global longitudinal strain and increases in arterial stiffness parameters among diabetic patients with a vitamin D deficiency, when compared to healthy controls, suggest that these measurements may serve as early indicators of myocardial and endothelial dysfunction. By monitoring serum 25(OH)D3 levels alongside indices of arterial stiffness and GLS, it may be possible to identify high-risk patients with type 2 diabetes mellitus prior to the emergence of overt cardiovascular disease. Although these associations are persuasive, there is a need for further high-quality, large-scale research to determine whether vitamin D deficiency is causally linked to the progression of vascular disease or if it merely reflects a general deterioration in health status. Future studies should incorporate adequate sample sizes and proper adjustments for confounding variables to effectively address this crucial question.

### Study Limitations

When interpreting the findings of this study, several limitations must be taken into account. Firstly, the cross-sectional design limits the ability to establish causal relationships among vitamin D deficiency, arterial stiffness, and myocardial dysfunction in patients with T2DM. To confirm temporal associations and causality, longitudinal studies are necessary. Secondly, the relatively modest sample size may restrict the generalizability of the results and diminish the statistical power to identify smaller effect sizes or interactions between variables. Larger, multi-center studies are warranted to validate these findings in more diverse populations. Thirdly, unmeasured confounding factors—such as physical activity levels, dietary patterns, and sun exposure—might have affected serum 25(OH)D3 levels and vascular function, potentially introducing a bias into the results. Fourthly, although LV GLS serves as a sensitive marker for subclinical myocardial dysfunction, we did not incorporate other advanced imaging modalities (for example, cardiac MRI or speckle-tracking for diastolic strain parameters) that could have offered a more thorough evaluation of the cardiac structure and function. A notable limitation is the age disparity between groups, with T2DM patients being older on average. While age did not emerge as an independent predictor in our multivariate analysis, it may still contribute to the observed differences in vascular parameters, as aging is associated with progressive endothelial dysfunction and reduced vitamin D synthesis. Sensitivity analyses in age-matched subgroups could be explored in larger studies.

Finally, vitamin D levels were measured at a single time point, which may not provide an accurate representation of the long-term vitamin D status or its association with chronic cardiovascular changes.

## 5. Conclusions

This study demonstrates that patients with type 2 diabetes mellitus without a clinically overt cardiovascular disease show marked elevations in arterial stiffness indicators (IMT and PWVao), decreased serum levels of 25(OH)D3, and a diminished left ventricular global longitudinal strain in comparison to healthy control subjects. The presence of a significant inverse correlation between serum 25(OH)D3 levels and the parameters of arterial stiffness underscores the possible involvement of vitamin D deficiency as a factor contributing to the initial vascular alterations observed in T2DM. Therefore, targeting vitamin D3 deficiency may be relevant not only for metabolic health but also as an early intervention to reduce the cardiovascular risk in this population. Furthermore, our findings suggest that a heightened arterial stiffness correlates with the detrimental remodeling of the left ventricle, thereby strengthening the notion of vascular–ventricular coupling in the development of diabetic cardiomyopathy.

These results highlight the clinical relevance of the early screening for arterial stiffness, myocardial strain abnormalities, and vitamin D deficiency in T2DM patients. Such measures could help identify individuals at a higher risk for future cardiovascular events before the onset of an overt disease.

Further prospective, large-scale studies are needed to determine whether the correction of vitamin D deficiencies could mitigate the arterial stiffness progression and improve cardiovascular outcomes in this high-risk population.

## Figures and Tables

**Table 1:** Characteristics of all patients in study (*n* = 120).

Variable	Healthy N = 55 ^1^	T2DM N = 65 ^1^	*p*-Value ^2^
GENDER			0.10
Female, n	25 (45%)	20 (31%)	
Male, n	30 (55%)	45 (69%)	
AGE, years	57 (52, 63)	64 (60, 69)	<0.001
Active smoking			0.3
No	31 (56%)	43 (66%)	
Yes	24 (44%)	22 (34%)	
BMI, kg/m^2^	24.8 (23.6, 27.3)	29.0 (26.3, 33.2)	<0.001
SBP, mmHg	130 (120, 132)	135 (120, 150)	<0.001
DBP, mmHg	82 (70, 84)	80 (70, 90)	0.3
HR, beats/min	69 (67, 82)	70 (64, 75)	0.2
Hb, g/dL	13.60 (12.30, 14.60)	14.30 (13.40, 15.20)	0.011
FPG, mg/dL	100 (94, 104)	104 (96, 118)	0.004
HbA1c, %	5.30 (5.20, 5.40)	7.90 (7.40, 8.30)	<0.001
Creatine, mg/dL	0.90 (0.80, 1.06)	1.02 (0.85, 1.17)	0.013
TG, mg/dL	116 (97, 135)	132 (95, 197)	0.035
LDL-c, mg/dL	86 (76, 106)	101 (82, 138)	0.015
25 (OH)D (ng/mL)	24 (23, 32)	19 (16, 24)	<0.001
Vitamin D deficit			<0.001
No	53 (96%)	28 (43%)	
Yes	2 (3.6%)	37 (57%)	

^1^ n (%); median (Q1, Q3); ^2^ Pearson’s Chi-squared test; Mann–Whitney U test; BMI, body mass index; SBP, systolic blood pressure; DBP, diastolic blood pressure; HR, heart rate; FPG, fasting plasma glucose; HbA1c, glycated hemoglobin; HB, hemoglobin; eGFR, estimated glomerular filtration rate; TC, total cholesterol; HDL-c, high-density lipoprotein cholesterol; LDL-c, low-density lipoprotein cholesterol; TGs, triglycerides; 25(OH)D3, 25-hydroxyvitamin D. Mean (SD) analyzed employing t-test, absolute frequency (%), analyzed employing Chi-square test.

**Table 2:** Echocardiographic and arterial stiffness parameters.

Variable	Healthy N = 55 ^1^	T2DM N = 65 ^1^	*p*-Value ^2^
LAD, cm	4.10 (3.80, 4.30)	3.90 (3.70, 4.00)	<0.001
IVS, mm	0.90 (0.90, 1.00)	1.10 (0.99, 1.20)	<0.001
LV_EDD, mm	4.50 (4.30, 4.70)	4.69 (4.46, 5.00)	0.002
LV_PW, mm	1.00 (0.90, 1.00)	1.10 (1.00, 1.16)	<0.001
LV_EDV, mL	86 (82, 90)	90 (84, 95)	<0.001
LV_ESV, mL	32.0 (31.0, 34.0)	38.0 (33.0, 42.0)	<0.001
LV_EF, %	55.0 (55.0, 55.0)	55.0 (50.0, 55.0)	0.080
LV_area	24.0 (22.0, 24.0)	21.0 (18.0, 26.0)	0.009
LAV, mL	36 (34, 38)	39 (33, 42)	0.023
E-wave, cm/s	0.80 (0.72, 0.82)	0.83 (0.72, 0.90)	0.004
A-wave, cm/s	0.52 (0.46, 0.60)	0.60 (0.52, 0.70)	<0.001
PA_SP	24.0 (22.0, 26.0)	28.0 (25.0, 32.0)	<0.001
e’, cm/s	6.50 (5.80, 7.20)	6.40 (5.80, 7.30)	0.9
E/e’ ratio	7.50 (7.00, 8.00)	11.80 (10.40, 12.40)	<0.001
LV_GLS, %	−20.20 (−21.40, −19.40)	−17.10 (−18.80, −16.00)	<0.001
IMT, mm	0.60 (0.50, 0.70)	1.10 (0.90, 1.30)	<0.001
PWVao, m/s	7.90 (7.40, 8.20)	10.10 (9.20, 10.80)	<0.001

^1^ Median (Q1, Q3); ^2^ Mann–Whitney U test; LAD, left atrium diameter; IVS, interventricular septum; LV_PW, left ventricular posterior wall; LV_EDD, left ventricular end-diastolic diameter; LV_EDV, left ventricular end-diastolic volume; LV_ESV, left ventricular end-systolic volume; LV_EF, left ventricular ejection fraction; LAV, left atrial volume; PA_SP, pulmonary artery systolic pressure; LV_GLS, left ventricular global longitudinal strain; IMT, intima–media thickness; and PWVao, pulse wave velocity. Mean (SD) analyzed employing *t*-test.

**Table 3:** Multivariate logistic regression of factors associated with arterial stiffness (PWV and IMT).

Variable	β	±SE	95% CI for OR	*p*-Value
Hypertension	0.786	1.266	0.10, 22.5	0.054
Diabetes	4.830	1.332	2.53; 8.10	0.001
Vitamin D deficit	2.402	1.187	0.28; 5.03	0.004

## Data Availability

The datasets are private, but de-identified data may be provided upon request from Florina Buleu.
